# Molecular interactions between metformin and *D*-limonene inhibit proliferation and promote apoptosis in breast and liver cancer cells

**DOI:** 10.1186/s12906-024-04453-x

**Published:** 2024-05-06

**Authors:** Elsayed I. Salim, Mona M. Alabasy, Eman M. El Nashar, Norah S. Al-Zahrani, Mohammed A. Alzahrani, Zihu Guo, Doha M. Beltagy, Mohamed Shahen

**Affiliations:** 1 Department of Zoology, Research Lab of Molecular Carcinogenesis, Faculty of Science, Tanta University, Tanta, 31527 Egypt; 2 Department of Anatomy, College of Medicine, King Khalid University, Abha, 62529 Saudi Arabia; 3 Department of Clinical Biochemistry, College of Medicine, King Khalid University, Abha, 62529 Saudi Arabia; 4 Internal Medicine Department, College of Medicine, King Khalid University, Abha, 62529 Saudi Arabia; 5 College of Life Science, Center of Bioinformatics, Northwest A and F University, Yangling, Shaanxi 712100 China; 6 Biochemistry Department, Faculty of Science, Damanhour University, Damanhour, Egypt

**Keywords:** Metformin, *D*-limonene, HepG2, MCF-7, Drug–target–pathway network, Gene analysis

## Abstract

**Background:**

Cancer is a fatal disease that severely affects humans. Designing new anticancer strategies and understanding the mechanism of action of anticancer agents is imperative.

**Hypothesis/Purpose:**

In this study, we evaluated the utility of metformin and *D*-limonene, alone or in combination, as potential anticancer therapeutics using the human liver and breast cancer cell lines HepG2 and MCF-7.

**Study design:**

An integrated systems pharmacology approach is presented for illustrating the molecular interactions between metformin and *D*-limonene.

**Methods:**

We applied a systems-based analysis to introduce a drug–target–pathway network that clarifies different mechanisms of treatment. The combination treatment of metformin and *D*-limonene induced apoptosis in both cell lines compared with single drug treatments, as indicated by flow cytometric and gene expression analysis.

**Results:**

The mRNA expression of *Bax* and *P53* genes were significantly upregulated while *Bcl-2*, *iNOS,* and *Cox-2* were significantly downregulated in all treatment groups compared with normal cells. The percentages of late apoptotic HepG2 and MCF-7 cells were higher in all treatment groups, particularly in the combination treatment group. Calculations for the combination index (CI) revealed a synergistic effect between both drugs for HepG2 cells (CI = 0.14) and MCF-7 cells (CI = 0.22).

**Conclusion:**

Our data show that metformin, *D*-limonene, and their combinations exerted significant antitumor effects on the cancer cell lines by inducing apoptosis and modulating the expression of apoptotic genes.

**Supplementary Information:**

The online version contains supplementary material available at 10.1186/s12906-024-04453-x.

## Introduction

Cancer is the leading cause of death worldwide, accounting for an estimated 9.6 million deaths in 2018 [1]. Cancer arises from the transformation of normal cells into tumor cells in a multi-stage process. The most common cancers are breast, prostate, lung, colorectal, cervical, stomach, liver, endometrial, ovarian, and thyroid [2, 3].

Breast cancer is the most common cancer in both sexes and the second leading cause of cancer mortality worldwide [4]. Breast cancer is highly heterogeneous encompassing a group of genetically and epigenetically distinct diseases exhibiting diverse clinical features [5]. The development of breast cancer is a multi-step process involving multiple cell types, and its prevention remains challenging [3]. One of the best prophylactic approaches is early diagnosis. Also, the discovery of breast cancer stem cells has helped us understand the development of the cancer and the behavior of many genes involved in its initiation and progression. Currently, many drugs are available for the chemoprevention of breast cancer, while natural products have recently been developed to improve the quality of life of patients and improve therapy [3]. A large proportion of current knowledge on breast carcinomas are derived from in vivo and in vitro studies performed using breast cancer cell lines, given that they could provide an unlimited source of homogenous self-replicating materials using simple yet standard media and approaches. Birnbaum et al. grouped 27 breast cancer cell lines into luminal, basal and mesenchymal subtypes [6]. Riaz et al. characterized 5 subtypes, i. e., luminal, luminal-HER2 + , ER-negative-HER2 + , basal, normal-like, among 51 breast cancer cell lines using a panel of 496 genes identified [7]. MCF-7 is a commonly used breast cancer cell line, it is ER-positive and progesterone receptor (PR)-positive and belongs to the luminal A molecular subtype. MCF-7 is a poorly-aggressive and non-invasive cell line, normally being considered to have low metastatic potential [8].

Although hepatocellular carcinoma (HCC) is only the seventh most common cancer in the world, it is the fourth leading cause of cancer mortality in both sexes, responsible for more than half a million deaths annually [9]. The major risk factors for HCC are chronic infections by hepatitis B and C viruses, and cirrhosis [10]. The epidemiology of HCC significantly varies by demography (age, gender, race/ethnicity) as well as geography. Recently, some major breakthroughs in the treatment of advanced HCC have been made [11].

The search for new therapeutics active against several types of cancers has become one of the most interesting challenges in the research on natural products. Plants have contributed greatly to the development of sophisticated traditional medicine systems, especially those with a long history in the treatment of cancer. Reports on the use of herbs are as old as humanity itself, demonstrating the potent medicinal and antitumor effects of plant-derived essential oils on different kinds of cancers [12].

Metformin is an oral, safe, biguanide antidiabetic drug derived from the herb *Galega officinalis* [13]*.* It is considered the first-line therapy for patients with type II *diabetes mellitus* and acts by reducing plasma glucose and lipids. The anticancer effects of metformin are associated with both direct and indirect actions of the drug. The indirect, insulin-dependent effects of metformin are mediated by the ability of adenosine monophosphate-activated protein kinase (AMPK) to inhibit the transcription of key gluconeogenesis genes in the liver, stimulating glucose uptake in muscles and reducing fasting blood glucose and insulin [14]. Since insulin has mitogenic and pro-survival properties, metformin may exert anticancer activity by lowering insulin. Tumor cells often express high levels of the insulin receptor, indicating a sensitivity to the growth-promoting effects of the hormone [15]. The direct, insulin-independent effects of metformin originate from liver kinase B1-mediated activation of AMPK and a reduction in mammalian target of rapamycin (mTOR) signaling and protein synthesis in cancer cells. AMPK impacts mTOR via phosphorylation and activation of the tumor suppressor protein tuberin, which negatively regulates mTOR activity. mTOR is a key integrator of growth factor and nutrient signals and a critical mediator of the phosphatidylinositol-3-kinase/protein kinase B/Akt signaling pathway, one of the most frequently dysregulated molecular networks in human cancer [16].

*D*-limonene (1-methyl-4-isopropyl-cyclohexene), a monocyclic monoterpene with a lemon-like odor, is a major ingredient in several citrus oils. It has low toxicity, pharmacological effects, and good tolerance, with no carcinogenicity in mice and rats. Its antitumor effects against numerous cancers, including HCC and breast cancer, have been reported [17]. *D-*limonene induces phase I and phase II carcinogen-metabolizing enzymes (cytochrome p450), which convert carcinogens into lesser toxic forms and prevent their interaction with DNA. It also inhibits carcinogenesis by inhibiting cell proliferation, enhancing apoptosis, and blocking oncogene expression [18]. Besides, it exhibited antioxidant and radical scavenging activities in several model systems and showed cytotoxicity against HCC [19] and human breast adenocarcinoma cells [20].

In this study, we investigated the effects of metformin in combination with *D*-limonene on the proliferation and apoptosis of HCC and breast cancer cells. We utilized the systems pharmacology approach to reveal the therapeutic mechanism of metformin and *D*-limonene. The Kyoto Encyclopedia of Genes and Genomes (KEGG) is a comprehensive database of known molecular interaction networks that are usually used to understand biological pathways and systems [21]. The database of drugs was screened out and targets were identified. Within the targets, we built a compound–target interaction using bioinformatic and pharmacological aspects. Subsequently, the targets were exploited to search the corresponding pathways in the KEGG database (http://www.genome.jp/kegg/) and the Database for Annotation, Visualization, and Integrated Discovery (DAVID, https://david.ncifcrf.gov/summary.jsp). We established a monolithic pathway to clarify the molecular pathogenesis at a systematic level. These results can help us understand the mechanism of the therapeutic combination against HCC and breast cancer.

## Materials and methods

### Network construction and topological analysis

To explain the reaction mechanisms of metformin and D-limonene as natural anticancer drugs, we constructed two types of networks: the compound–target (C–T) network and the target–pathway (T–P) network. The target name was entered in the Comparative Toxicogenomic Database (http://ctdbase.org/) and the data were input in Microsoft Excel. The Cytoscape software ver. 3.7.2. was used to generate all the displayed networks [22]. The quantitative properties of networks were analyzed using two plugins: Network Analyzer and CentiScaPe software, ver. 1.2 [23]. We used nodes to represent the compounds and targets, and lines between nodes represented interactions. The degree of a node was the number of edges linked with the node; it was used to determine the size of each node and to evaluate the characterization of different drug treatments from a network perspective, ensuring that the graph would be obvious. Nodes with the highest connectivity and global centrality, measured by degree and betweenness, were considered the most influential nodes in the whole network. In systems pharmacology a network, which consists of nodes and edges (connections between nodes), is a mathematically expressed, computationally measurable representation of the various interactions that underlie the intricate biological systems [24]. In order to explore comprehensively the interrelationship among metformin and D-limonene and potential targets which will help us to select out those particular targets genes related to breast cancer. In the resultant networks, compound predicted targets genes and enrichment functions diseases were illustrated via nodes; while edges indicated the interactions between them [25].

### Gene Ontology (GO) and KEGG pathway enrichment analyses

Making a formula for the treatment of a specific disease is usually easier than figuring out how it works. To further investigate the mechanisms of metformin and *D*-limonene, GO and KEGG enrichment analyses were performed in DAVID (https://david.ncifcrf.gov/). The GO analysis detects the biological properties of target genes [26].

### Cell culture

The hepatocellular carcinoma and breast cancer cell lines, HepG2 and MCF-7, were used to evaluate the antitumor activity of metformin and *D*-limonene, either alone or in combination. The obtained half-maximal inhibitory concentration (IC_50_) was tested on a normal cell line (WI-38) to confirm the cytotoxicity of the dose. HepG2, MCF-7 and WI-38 cells were purchased from the Cell Culture Department of the Holding Company for Biological Products & Vaccines (VACSERA), Dokki—Giza, Egypt. Informed consent was obtained by the provider from all subjects and/or their legal guardian(s). HepG2 and MCF-7 cells were cultured in (RPMI) with L-Glutamine (Lonza, BioWhittaker), WI-38 cells were cultured in Dulbecco's modified Eagle medium culture medium glucose with L- glutamine (DMEM, Lonza, USA) and supplemented with 10% Fetal Bovine Serum (FBS), (100U) 20 μg/ml penicillin and 100 μg/ml streptomycin. Incubation was carried out at 37 °C with an atmosphere of 5% CO2. The cellular viability was checked using trypan blue. Metformin and D-limonene were dissolved in Dimethyl Sulfoxide (DMSO) from Sigma-Aldrich, USA with concentration 500 ug/ml for each compound.

All human cell lines used in this experiment have been approved by the appropriate ethics committee and have therefore been performed in accordance with the ethical standards laid down in the 1964 Declaration of Helsinki, and its later amendments. The Research Ethical Committee (REC) and The Institutional Animal Care Committee at Tanta University's Faculty of Science's Zoology Department approved the experimental protocol under approval No.: REC/IACUC/SCI/TU/0173. There were no humans or animals involved in this investigation.

### Cytotoxicity assay

The MTT (3-(4,5-Dimethylthiazol-2-yl)-2,5-diphenyltetrazolium bromide) colorimetric assay (Cell Titer 96 Aqueous One Solution Cell Proliferation Assay, Promega, USA) was used to determine cytotoxicity. Briefly, triplicates of 1 × 10^4^ cells/well of HepG2 and MCF-7 were treated with 200 µL/well of the metformin and *D*-limonene working solution prepared in RPMI. Metformin was added to the media at concentrations 200, 100, 50, 25, 12.5, and 0 µg/ml; *D*-limonene was added at concentrations 100, 50, 25, 12.5, 6.25, and 0 µg/ml. All plates were incubated in a humidified 5% CO_2_ incubator at 37 °C for 24 h or 48 h. MTT assay was performed according to the method developed by Mosmann with some modifications [27]. The cell growth in each plate after 24 or 48 h was measured at 570 nm using a microplate reader (LMR-9602, U.S.A) and the IC_50_ concentrations were calculated.

### Drug combination study analysis

According to the IC_50_ doses of metformin and *D*-limonene obtained from the MTT assay on HepG2 and MCF-7 cells, a combined-treatment regimen was designed in the ratios 1:1, 1:2, 2:1, 1:4, 4:1, 1:9, and 9:1 [28]. The constant ratio of 2:1 (metformin:*D-*limonene), was deduced (data not shown) as the most effective dose, which was finally applied to HepG2 and MCF-7 cells. All the plates were incubated in humidified 5% CO_2_ at 37 °C for 24 or 48 h.

The cytotoxicity of the combined treatment on HepG2 and MCF-7 cells was evaluated by the combination index (CI) using the Chou and Martin method on the CompuSyn software for drug combinations for general dose–effect analysis (ComboSyn, Inc. Paramus, NJ 2007, USA) [www.combosyn.com]. The CI values indicate a synergistic effect when < 1, an antagonistic effect when > 1, and an additive effect when equal to 1 [29].

## Apoptosis detection methods by flow cytometry

### Annexin V-FITC/PI staining assay

After treating HepG2 and MCF-7 cells with the IC_50_ doses of metformin, *D*-limonene, or their 2:1 combination, cellular apoptosis levels were measured quantitatively by determining the amount of cell surface phosphatidylserine using the Annexin V-FITC/PI apoptosis detection kit (BD Pharmingen). The cells were analyzed using a fluorescence-activated cell sorting (FACS) caliber flow cytometer (Becton Dickinson, Sunnyvale, CA, USA), with emission filters of 488 nm [30].

### Cell cycle analysis by flow cytometry

After treating HepG2 and MCF-7 cells with the IC_50_ doses of metformin, *D*-limonene, or their combination, cells were harvested and incubated with propidium iodide for 15 min before being analyzed on a FACS caliber flow cytometer. Cell cycle distribution was analyzed using the Modifit’s program (Becton Dickinson). The staining of mammalian DNA for flow cytometry was performed according to Darzynkiewicz, who used stoichiometric dyes that bind in proportion to the amount of DNA present in the cell. Since cells that are in the S phase will have more DNA than cells in the G1 phase, the fluorescent dye appears more intense in the S phase than in the G1 phase. The cells in G2 will be approximately twice as bright as the cells in G1 [31].

## Determination of antioxidative stress markers

### DPPH radical scavenging assay

2,2-diphenyl-1-picrylhydrazyl (DPPH) was purchased from Sigma Chemical Co. (USA). The free radical scavenging ability of metformin and *D-*limonene was tested by the DPPH radical scavenging assay, as described previously [32]. The ability of the drugs to donate their hydrogen atom was determined by the decolorization of a methanol solution of DPPH. DPPH produces a violet/purple color in methanol, which fades to shades of yellow in the presence of antioxidants. A solution of 0.1 mM DPPH in methanol was prepared, and 1 mL of this solution was mixed with 3 mL of metformin and *D*-limonene solution at different concentrations (1, 5, 10, 25, 50, and 100 μg/mL). The reaction mixture was vortexed thoroughly and left in the dark for 30 min. The absorbance of the mixture was measured spectrophotometrically at 517 nm. Ascorbic acid was used as a reference drug. Percentage DPPH radical scavenging activity was calculated by the following equation:

$$\%\;\mathrm{DPPH}\;\mathrm{radical}\;\mathrm{scavenging}\;\mathrm{activity}\:=\;\left[\left({\mathrm A}_0-{\mathrm A}_1\right)/{\mathrm A}_0\right]\;\times\;100$$

Where A_0_ is the absorbance of the control and A_1_ is the absorbance of the drugs/standard. The percentage of inhibition was plotted against concentration, and from the graph, the IC_50_ was calculated. The experiment was repeated thrice at each concentration.

### Determination of reduced glutathione concentration (GSH)

Reduced glutathione concentration was colorimetrically determined according to the method described previously [33]. The reduced glutathione determination is based on the reduction of the disulfide chromogen 5,5´-dithiobis (2-nitrobenzoic acid) by GSH to produce an intensely yellow compound. The absorbance of the reduced chromogen was spectrophotometrically measured at 412 nm and is directly proportional to the GSH concentration. *Bax, Bcl2, P53, PTGS2 (COX-2),* and *iNOS* gene expression.

### RNA extraction

Total RNA was isolated from MCF-7, HepG2, and WI-38 cells using the Gene JET RNA Purification Kit (Thermo Scientific, USA), according to the manufacturer’s protocol. The purity and the concentration of the extracted RNA were measured on a Nanodrop spectrophotometer. RNA (1 μg) was reverse transcribed using the SensiFAST™ cDNA Synthesis Kit (Thermo Co, America). The produced cDNA was used as a template to determine the relative expression of *Bax, Bcl2, P53, PTGS2 (COX-2),* and *iNOS* genes using a real-time PCR system (Rotorgene 5plex, Germany) and specific primers (Supplementary Table 1). Glyceraldehyde 3-phosphate dehydrogenase was used as an internal control. The thermal cycling, melting curve analysis, and calculation of relative expression using 2^−ΔΔCt^ were done as previously described [34].

### Statistical analyses

All experiments were performed in triplicates; the data are presented as means ± standard deviation. The statistical analysis and plotting of data were done using GraphPad Prism software ver7 San Diego, CA 92108. *P* < 0.05 was considered statistically significant.

## Results

### Drug targets and analysis

**Fig. 1:**
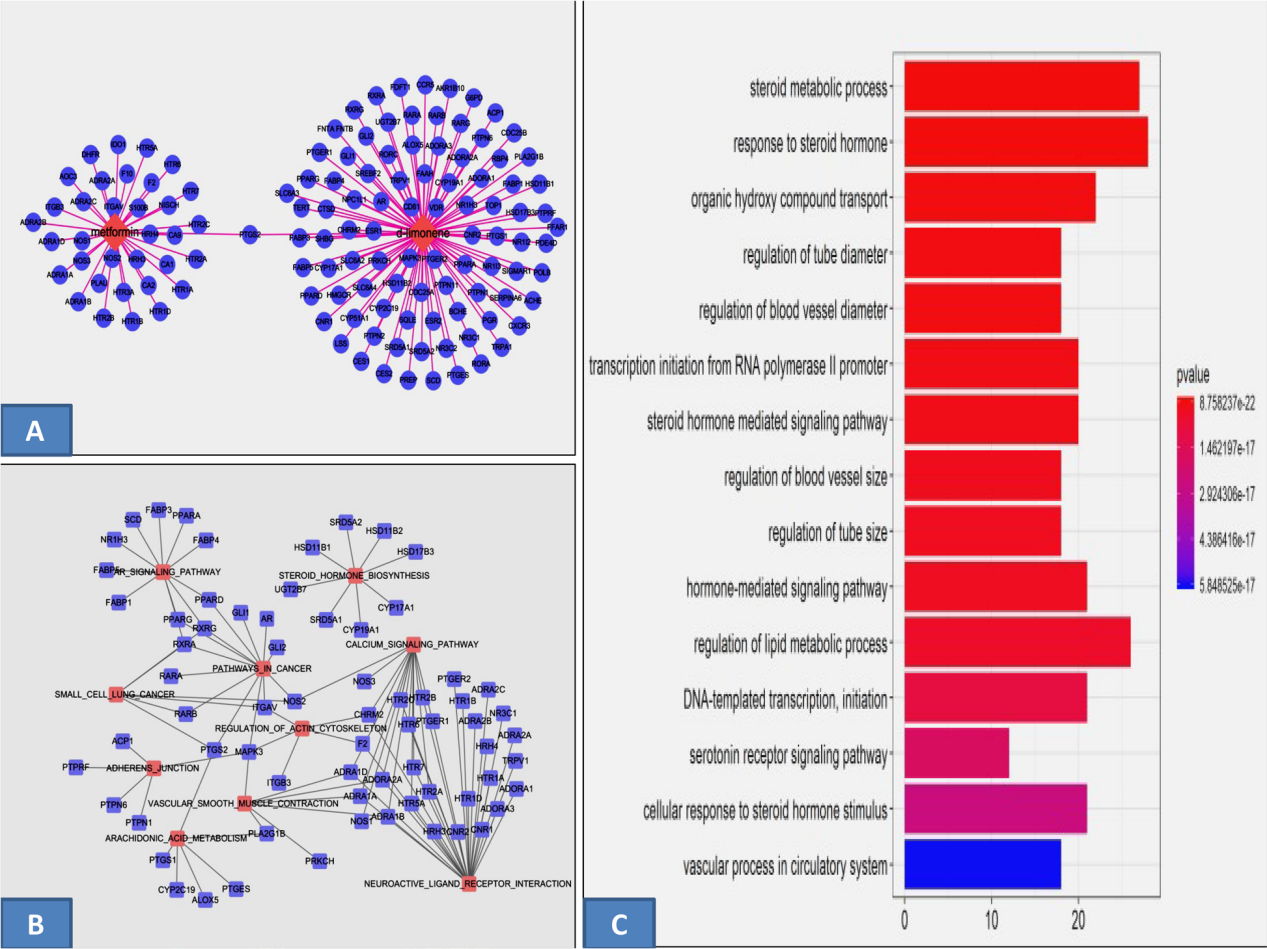
**a** The compound–target network of metformin and D-limonene. The blue nodes represent target genes and the pink nodes represent compounds, the edges are the interactions of targets and their related pathways. **b** The target–pathway network of metformin and D-limonene. The blue nodes represent target genes and the pink nodes represent pathways, the edges are the interactions of targets and their related pathways. **c** The Gene Ontology enrichment of therapy target genes. Y-axis represents significantly enriched biological process categories relative to target genes and the X-axis shows the counts of targets

In this study, we proposed an integrated platform of systems pharmacology combining target fishing and network pharmacology to dissect the molecular mechanisms of metformin and *D*-limonene. We found 35 and 93 predicted targets for metformin and *D*-limonene, respectively, using DAVID to systematically analyze their biological processes (Fig. 1A). To investigate whether metformin and *D*-limonene had pathological effects, we evaluated their impact on physiological processes through GO analysis. Figure 1B, C lists the 15 foremost GO terms; these vital targets have a very strong correlation with physiological mechanisms, such as regulation of programmed cell death and cell proliferation, and response to steroid hormones.

### The T–P network

To elucidate the different disease-related pathways that metformin and *D*-limonene might be influencing the most, nine significantly overrepresented pathways were extracted from the KEGG database (Supplementary Table 2). The T–P network indicated that the targets of these drugs were involved in different pathways, such as neuroactive ligand–receptor interactions, calcium signaling, steroid hormone biosynthesis, peroxisome proliferator-activated receptor (PPAR) signaling, cancer, and vascular smooth muscle contraction. All the potential protein targets were mapped onto the signaling pathways, and a bipartite network of targets and their pathways was constructed to explore the therapeutic effects of the drugs in various diseases (Supplementary Table 3).

### The antitumor effect of metformin and *D*-limonene on HepG2 and MCF-7 cells

**Fig. 2:**
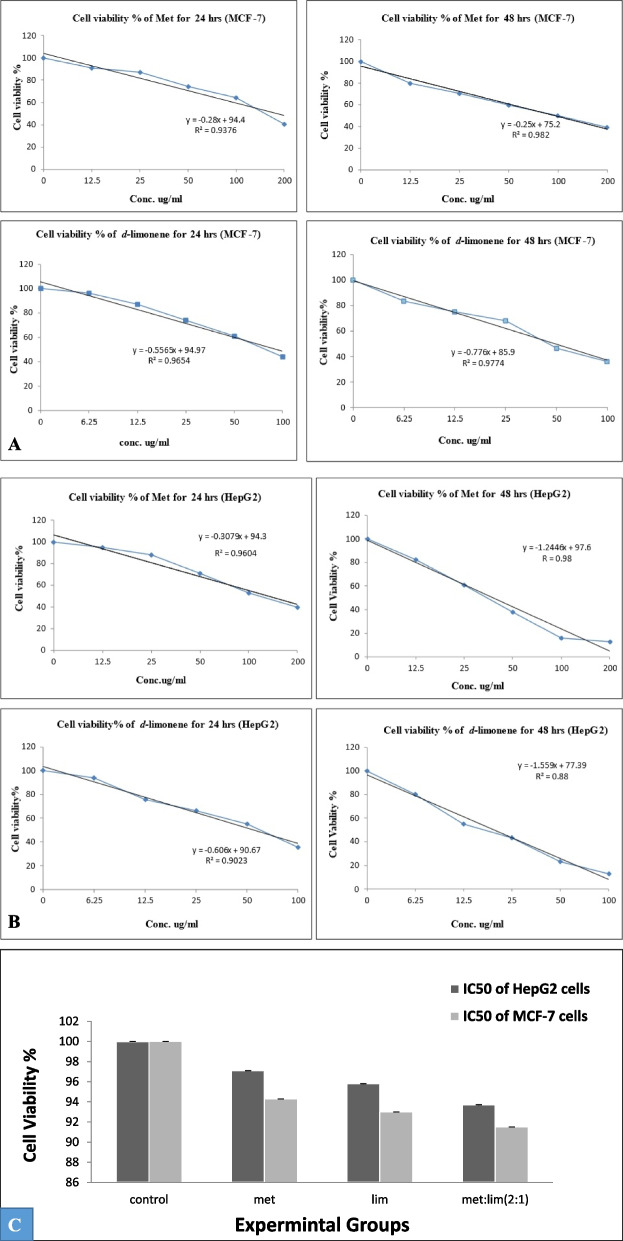
**a** The antitumor effect of metformin and *D*-limonene on HepG2 cells after incubation for 24 and 48 h. **b** The antitumor effect of metformin and *D*-limonene on MCF-7 cells after incubation for 24 and 48 h. **c** The cytotoxicity of IC_50_ doses of metformin and *D*-limonene toward the normal cell line WI-38 after incubation for 48 h. The percentage of viable cells was measured by the MTT assay. met: metformin, lim: *D*-limonene

From Figs. 2A and 2B, the IC_50_ of metformin and *D*-limonene in HepG2 cells was 144 and 67 µg/mL after 24 h, 38.4 and 17.7 µg/mL after 48 h, respectively; in MCF-7 cells, the IC_50_ was 158.7 and 80.8 µg/mL after 24 h, 100.8 and 46.3 µg/mL after 48 h, respectively.

### The cytotoxicity of metformin and *D*-limonene toward WI-38 cells

Figure 2C shows that the two compounds had a minimal cytotoxic effect on WI-38 (a normal cell line) when used at doses corresponding to the 48 h-IC_50_ values calculated for HepG2 and MCF-7 cells. The cell death did not exceed 10% of the total cells.

### The cell viability of HepG2 and MCF-7 cells after combined treatment

**Fig. 3:**
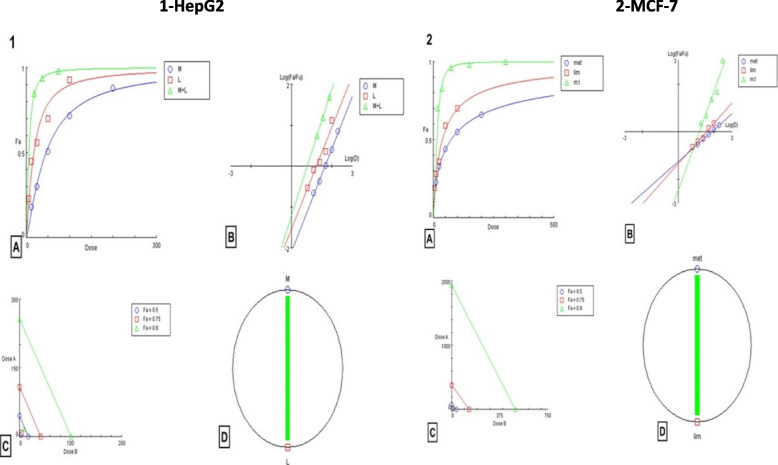
1) CompuSyn-generated graphics based on numerical data given in Supplementary Table 4-A for HepG2 cells. 2) CompuSyn-generated graphics based on numerical data given in Supplementary Table 3-B for MCF-7 cells. a: dose–effect curves. b: median-effect plots. c: isobologram for combo: (Met + lim [2:1]). d: polygonogram at Fa = 0.9

In this study, we combined metformin with *D-*limonene, for the first time as far as we know, in different volumes of constant IC_50_ concentrations to discover that a 2:1 ratio of metformin: *D*-limonene; 38.4 × 2 µg/mL: 17.7 µg/mL (for HepG2 cells) and 100.8 × 2 and 46.3 µg/mL (for MCF-7 cells), was optimal. Combined treatment in this ratio dramatically decreased cancer cell proliferation compared with single treatments. The present data show that metformin and *D-*limonene, alone or in combination, exerted a high antitumor effect on HepG2 and MCF-7 cells (Supplementary Table 4). By applying the CompuSyn software for CI, we demonstrated a very strong synergistic effect (CI < 1) upon combining the two compounds, which induced stronger activation of apoptosis and stronger inhibition of tumor cell growth in HepG2 and MCF-7 cells (Fig. 3). The CI for HepG2 cells was 0.14, and for MCF-7 cells was 0.22.

### DPPH radical scavenging activity of metformin and *D*-limonene

**Fig. 4:**
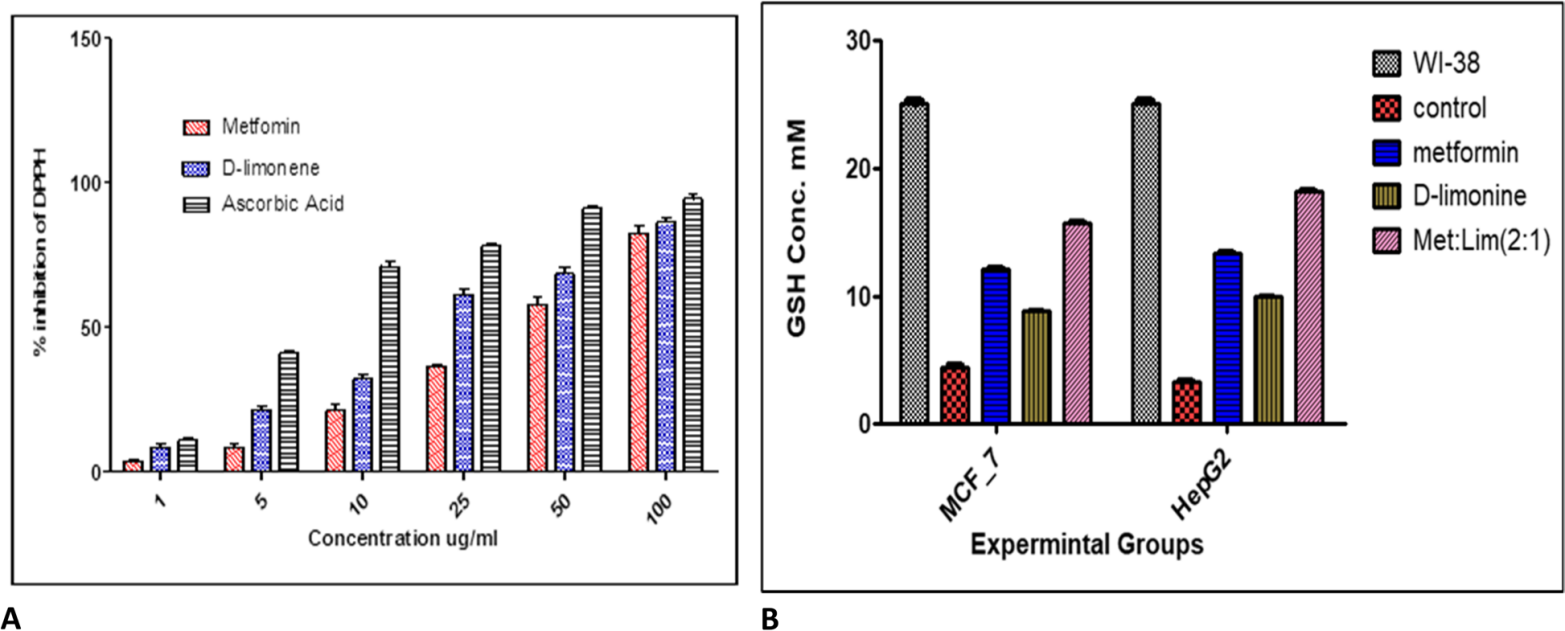
**a** DPPH free radical scavenging activity at different concentrations of metformin, *D*-limonene, and ascorbic acid. **b** GSH concentration in HepG2 and MCF-7 cells cultured in the basic medium as well as medium supplemented with metformin, *D*-limonene, and combination of both drugs (Met: Lim [2:1]) at IC_50_ dose levels

Our analyses revealed that the scavenging ability of metformin and *D*-limonene was higher than the model antioxidant substance ascorbic acid The percentage scavenging of DPPH free radical was found to be concentration-dependent – concentration of the compounds between 1–100 µg/mL greatly increased their inhibitory activity (Fig. 4A), with an IC_50_ value of 32.2 ± 1.23 and 18.5 ± 0.978 µg/mL for metformin and *D*-limonene, respectively, compared with 5.858 ± 0.767 µg/mL for ascorbic acid.

### Effect on GSH concentration

Treatment of HepG2 and MCF-7 cells with metformin, *D-*limonene, and their combination, at doses corresponding to their respective IC_50_ concentrations, induced a significant increase in GSH concentration compared with untreated cancerous cells (*P* < 0.05). Among the experimental groups, the highest GSH concentration was observed in cells subjected to the combined treatment (Fig. 4B).

### Detection of apoptosis by flow cytometry

**Fig. 5:**
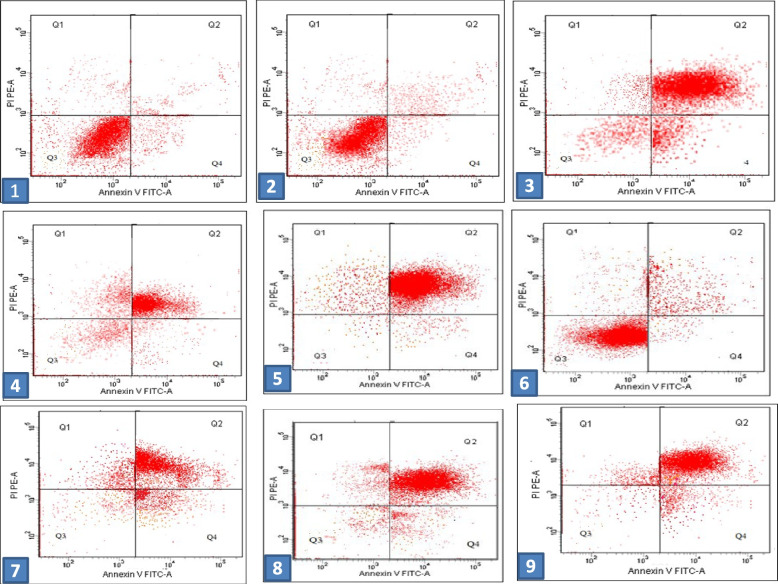
Dot plot flow cytometry analysis data. 1) Control untreated normal cells; 2) untreated HepG2 cells; 3) HepG2 cells treated with IC_50_ dose of *D-*limonene; 4) HepG2 cells treated with IC_50_ dose of metformin; 5) HepG2 cells treated with a combination of Met: Lim (2:1); 6) untreated MCF-7 cells; 7) MCF-7 cells treated with IC_50_ dose of* D-*limonene; 8) MCF-7 cells treated with IC_50_ dose of metformin; 9) MCF-7 cells treated with a combination of Met: Lim (2:1)

**Fig. 6:**
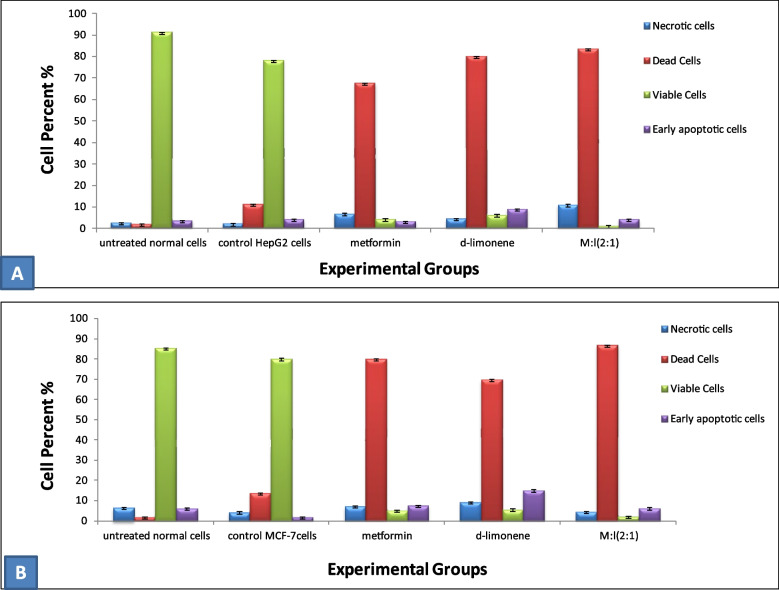
Flow cytometric analysis data showing average percentages of viable, necrotic, early, and late apoptotic **a** HepG2 cells and **b** MCF-7 cells treated with metformin, *D*-limonene, and a combination of both Met:Lim (2:1) compared with untreated normal cells

**Fig. 7:**
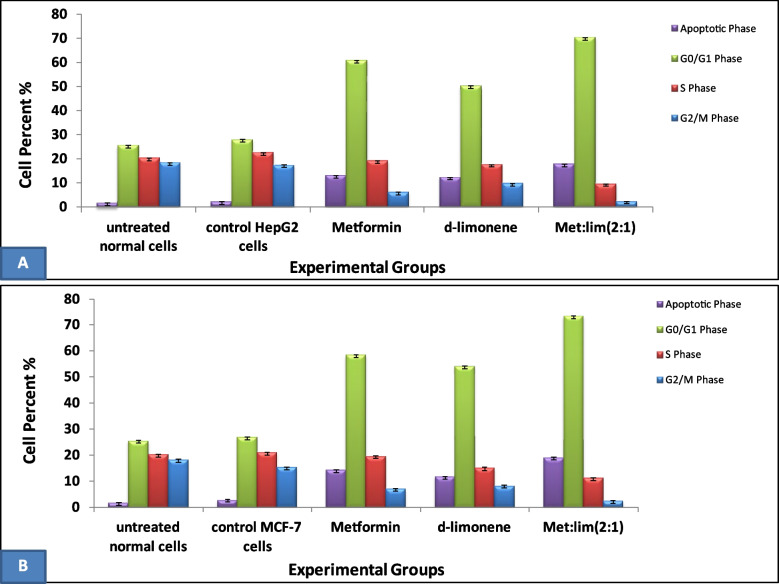
Flow cytometric analysis data showing average cell percentages in each phase of cell cycle stained with Propidium iodide for HepG2 cells **a** and MCF-7 cells **b**

**Fig. 8:**
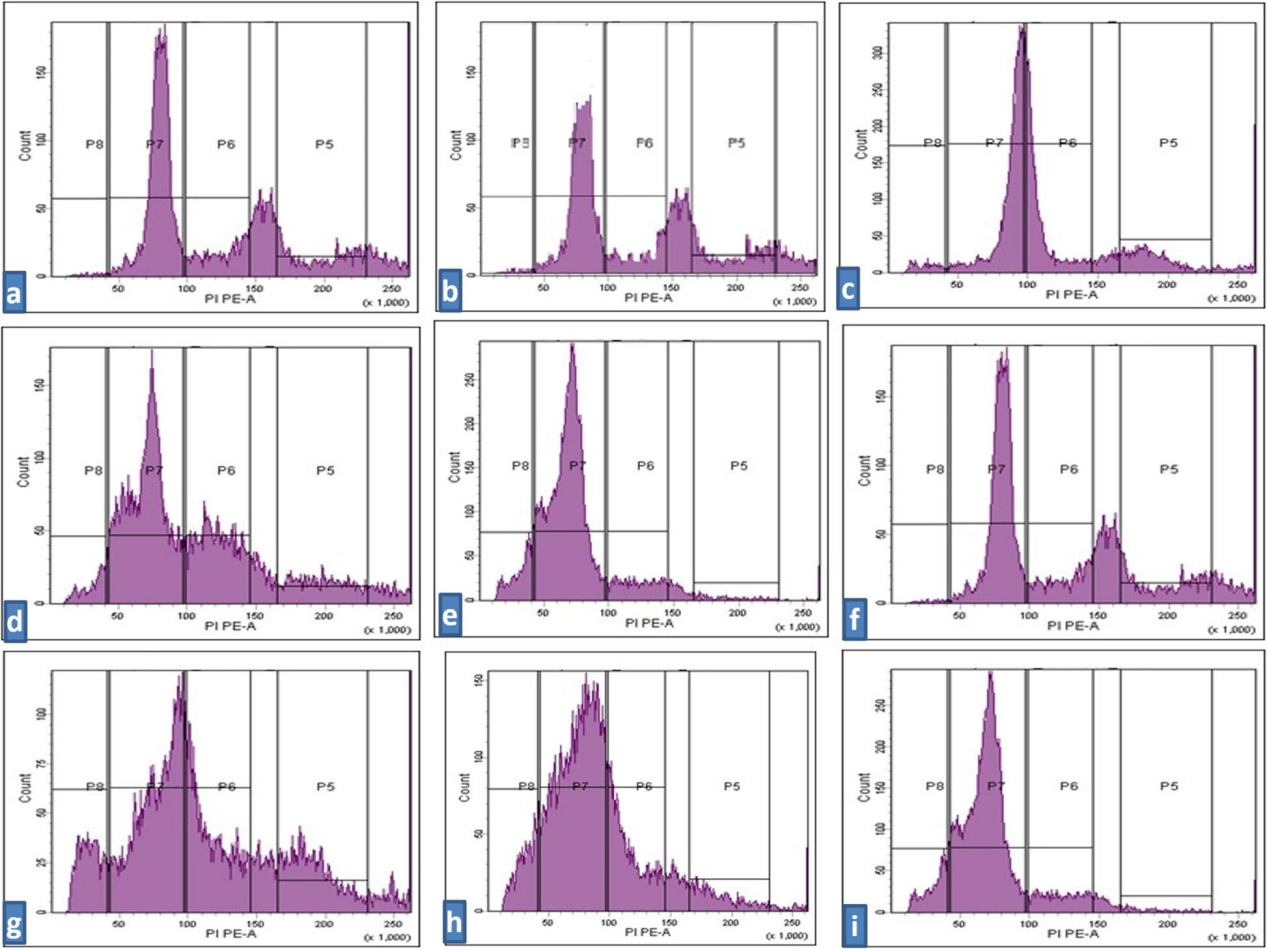
Flow cytometric histograms for cell cycle analysis stained with Propidium iodide showing the cell cycle phases P8 = Apoptosis; P7 = (G0/G1) the first gap phase; P6 = S phase; P5 = G2/M. show HepG2 cells treatment affected the cell cycle distribution and induced apoptosis. **a **Control untreated normal cells; **b **untreated HepG2 cells; **c** HepG2 cells treated with metformin; **d **HepG2 cells treated with *D*-limonene; **e** HepG2 cells treated with combination of Met: Lim (2:1); **f **untreated MCF-7 cells; **g **MCF-7 cells treated with metformin IC50; **h **MCF-7 cells treated with d-limonene IC50; **i** MCF-7 cells treated with combination of Met: Lim (2:1)

Flow cytometry was used to detect apoptosis and determine cell cycle stage in HepG2 and MCF-7 cells, treated with the respective IC_50_-dose of the two compounds alone or in combination, versus untreated control cells. The percentage of apoptotic HepG2 cells (both early and late stage) significantly increased in the treatment groups, with ratios of 67.5%, 79.7%, and 83.5% for metformin-, *D*-limonene-, and combination-treated cells, respectively, compared with the control cells (11.3%) (Fig. 5–2, 3, 4, 5). The combined treatment significantly induced the highest percentage of apoptosis in HepG2 cells (Fig. 6-A). Similarly, for MCF-7 cells, (Figs. 5–6, 7, 8), the combined treatment induced the highest percentage of apoptosis with a ratio of 86.9%, while metformin- and *D*-limonene-treated cells showed ratios of 80.1% and 69.8%, respectively. Untreated MCF-7 cells had an apoptotic ratio of just 13.7% (Fig. 6B).

### Cell cycle analysis by flow cytometry

In HepG2 cells, as shown in Fig. 7A, a significant G_0_/G_1_ phase arrest was observed which further increased in all the treated groups compared with the control group. The highest percentage of cells arrested in G_0_/G_1_ was seen in the combination-treated group (70.2%), followed by 60.2% and 50.2% in the metformin- and *D*-limonene-treated groups, respectively. Concomitantly, a significant decrease in the proportion of cells in the S phase was observed, from 22.5% in the control group to 19.2%, 17.6%, and 9.6% in the metformin-, *D-*limonene-, and combination-treated groups, respectively. Also, there were significantly fewer cells in the G_2_/M phase in the combination-treated group (2.4%) than in the metformin- (6.2%) and *D-*limonene-treated groups (9.8%), while the control group had 17.4% cells in this phase. The mean number of apoptotic HepG2 cells, represented by the sub-G_0_/G_1_ phase, were significantly higher in the treated groups compared with the control group. The combination-treated group had 17.7% apoptotic cells, the metformin-treated group had 13%, and the *D*-limonene-treated group had 12.4% (Fig. 8, a-e). The percentage of cells in all phases, except the G_2_/M, were higher in untreated HepG2 cells than in untreated normal cells (Fig. 7A).

The cell cycle analysis results for MCF-7 cells were similar (Fig. 8, f-I). The combination-treated group showed the highest percentage of cells arrested in G_0_/G_1_ (73.4%), followed by 58.5% and 54.2% in the metformin- and *D*-limonene-treated groups, respectively. Concomitantly, the combination-treated group showed the lowest percentage of cells in the S phase (11.3%), followed by 19.6% and 15.2% in the metformin- and *D-*limonene-treated groups, while 21.1% of cells in the control group were in this phase. Also, 2.4% of the cells in the combination-treated group were in the G_2_/M phase compared with 7.1%, 8.3%, and 15.4% for the metformin-, *D-*limonene-treated, and control groups, respectively. The combination-treated group had 19.1% apoptotic cells in contrast to 14.4% and 11.8% in metformin- and *D*-limonene-treated groups, respectively. The percentage of cells in all phases, except G_2_/M, was generally higher for the untreated MCF-7 cells than the untreated normal cells (Fig. 7B).

### Expression of the *Bax, Bcl2, P53, PTGS2 (COX2)* and *iNOS genes*

The quantitative PCR results for both HepG2 and MCF-7 cell lines revealed a significant downregulation (*P* ≤ 0.05) in the expression of *Bcl2, PTGS2* and *iNOS* genes whereas a significant upregulation in the expression of *Bax* and *P53* genes in the treated groups compared with the control group. Again, the combined-treatment group exhibited effects of the highest magnitude compared with other treatment groups.

## Discussion

The predicted targets are associated with a series of diseases, including different types of cancers. The drug–target network analysis revealed effective interaction between metformin and *D*-limonene through the *PTGS2* (*COX-2*) gene, which encodes a key enzyme that mediates prostaglandin synthesis and is involved in tumor invasiveness and angiogenesis [35]. Targeting this gene will be efficient because it will likely impact multiple nodes at the system level. *COX-2* is widely upregulated in many human cancers, including colorectal, prostate, breast, gastric, hepatic, lung, and head and neck cancers, as it is promoted by a variety of cytokines, growth factors, and tumor promoters [36]. More importantly, the novel potential pharmacological effects of metformin and *D*-limonene were illustrated. Also, among these targets, the inducible nitric oxide synthase (*iNOS*) or type II genes correlated with carcinogenic processes in liver and breast cancers play a central role in inflammation and expresses protective effects against detrimental damage [37].

Recent studies have shown the effect of metformin and *D*-limonene on cancer cells through GO analysis. All these physiological mechanisms such as regulation of programmed cell death and cell proliferation, and response to steroid hormones are interrelated to the pathogenesis of HCC and breast cancer. Several recent clinical studies demonstrated the antitumorigenic effect of metformin and *D*-limonene, with high cytotoxic selectivity, in different cancerous cells in vitro and in vivo [38]. Our results for *D*-limonene were similar to those of Hajizadeh et al. [39], who showed that 400 µM of the compound was cytotoxic to HepG2 and MCF-7 cancer cells. Das et al. [40] reported the IC_50_ of metformin in HepG2 cells to be 25 mM. Kuang et al. [41] reported similar results when they compared senescence due to oxidative stress in human periodontal ligament cells and WI-38 cells. Mahmoud et al. [42] studied the antioxidant, antibacterial, and antitumoral effects of *Cymbopogon citratus*, *Mentha longifolia*, and *Artemisia absinthium* essential oils on WI-38 cells. They reported very low cytotoxic effects of the herb oils on WI-38 cells in contrast to considerable cytotoxicity in MCF-7 and the colon cancer cell line HCT112.

Previous studies [43, 44] showed a synergistic anticancer effect against HepG2 and MCF-7 cells when metformin was combined with many natural extracts. Similarly, recent research [45] reported a synergistic effect against the same cell lines when *D*-limonene was combined with many natural products.

The strongest safe antioxidants are essential to prevent the free radical-mediated progression of disorders. They can either scavenge reactive oxygen/nitrogen species (ROS/RNS) to stop radical chain reactions (primary antioxidants or free radical scavengers) or inhibit the reactive oxidants from being converted into ROS/RNS (secondary or preventive antioxidants) [46]. This DPPH radical scavenging activity might confirm the hydrogen donating capacity of these compounds and explain their proposed ability to protect consumers from various free radical-related diseases. Here, we used the equation between weight and molecular weight, to convert the values of µM to µg/ ml by multiplying µM by molecular weight then divided by 1000, quantifying the values with µg/ ml and µM. Previous studies have demonstrated the efficiency of metformin and *D-*limonene in managing oxidative stress [47]. On the other hand, previous studies have also shown that metformin increased GSH levels in MCF-7 and HepG2 cells [48]. Furthermore, useful information [49] were provided about the antioxidant, antidiabetic, anticancer, and anti-inflammatory effects of *D-*limonene. Adhikary et al. [50] have previously studied the effect of *D*-limonene on HepG2 cells and showed that limonene expanded the rate of apoptotic cells up to 89.61%, by flow cytometry, and 48.2% by fluorescence microscopy.

Previous studies [51] have shown that these two compounds significantly enhanced cellular apoptosis in MCF-7 cells. The regulation of cell proliferation depends on a balance between cell division and death. In cancer cells, this balance swings toward proliferation because of a dysregulated cell cycle, making them immortal. Previous studies, including in vitro experiments, animal models, and epidemiological analyses, have shown that metformin and *D*-limonene inhibit tumor cell proliferation.

Previously, they were shown to inhibit HCC and breast cancer growth by arresting the tumor cell cycle in the G_0_/G_1_ phase and promoting apoptosis via the AMPK-dependent pathway [52]. Xiong et al. [53] showed that metformin arrested the cell cycle and induced apoptosis in HCC cells through an AMPK-independent pathway. In the present study, metformin arrested HCC and MCF-7 cells at the G_1_ phase of the cell cycle, and induced apoptosis, compatible with previous findings [54, 52]. Therefore, metformin may be useful to control the proliferation of HCC and MCF-7 cells. Several monoterpenes, including *D-*limonene, were also reported to act as antiproliferative agents against cancer cells by arresting cell growth, both in G_0_/G_1_ or G_2_/M phases [55]. Previous studies have demonstrated the antitumorigenic effects of *D*-limonene on cancer [18], and promoted the identification of proapoptotic, anti-inflammatory, antiproliferative, anti-invasive, and potential antiangiogenic activities of limonene by employing a dual reverse virtual screening protocol.

These data support our speculation that the treatment regimens trigger apoptosis by activating *p53* and *Bax*. Our results agree well with those of Hafidh et al. [56], who investigated the effect of natural drugs on tumor cells and reported a reduction in growth, as well as the synthesis of DNA, RNA, and gene expression in these cells.

The present study revealed that administration of the natural products metformin and *D-*limonene, either alone or in combination, reduced cell viability or induced apoptosis in HepG2 and MCF-7 cells, which was evident from the significant increase in the expression of *p53* and the proapoptotic *Bax* genes with a concomitant decrease in the anti-apoptotic *Bcl2* gene. Cells subjected to combined treatment showed the highest *Bax* and lowest *Bcl2* expression, indicating a higher apoptotic rate and synergism. These in vitro data confirmed our in silico iso bologram results and CI analysis for drug interaction, and together they indicate a strong synergistic effect between the two tested compounds. Our results are aligned with previous studies, which showed that metformin could induce apoptosis and inhibit the growth of hepatocellular and breast cancer cells [57], by downregulating *Bcl2* expression and upregulating *Bax* and *P53* expression. Limonene also displayed proapoptotic effects in the T24 human bladder [58] and HepG2 cancer cells by increasing *Bax* and decreasing *Bcl2* expression [59], indicating that the apoptosis-dependent anticancer effect of these natural products is not restricted to a single cancer type. *Bax* and *Bcl2* are known to be activated by the tumor suppresser *p53* during apoptosis [60]. The apoptosis induced by metformin and *D*-limonene, in this study, was associated with the downregulation of *PTGS2* (*COX2*) and *iNOS* genes, upregulation of *p53*, and an increased *Bax/Bcl2* ratio*.*

Moreover, the present study shows that the percentage of detectable *iNOS* mRNA significantly decreased in all the treated groups compared with the untreated MCF-7 and HepG2 cells, while it was 7.7-fold and tenfold higher, respectively, in these cells compared with the untreated controls. In agreement with these findings, Previous studies have shown that the expression of *p53* downregulates *iNOS* by inhibiting its promoter through a *p53*-dependent mechanism [61]. On the other hand, the *COX-2* gene is widely upregulated in many human cancers, including breast [62] and liver [63], indicating its role in promoting tumorigenesis. *COX-2* was found to promote cell proliferation and inhibit apoptosis by mediating the activation of downstream oncogenic pathways [64]. Our study showed a significant downregulation of *COX-2* in all treated groups compared with the untreated group of normal cells, thus proving the specific utility of metformin and *D-*limonene together in the treatment of cancer.

## Conclusion

In summary, the present work dissected the therapeutic poly-pharmacology mechanisms of metformin and *D-*limonene, which may provide a systematic strategy to study their mechanisms of action, while promoting novel drug discovery by their combination. Also, the potency of metformin, *D-*limonene, and their combination to inhibit HepG2 and MCF-7 cells in vitro is associated with apoptosis, which is indicated by increasing the expression of *p53* and the proapoptotic gene *Bax* and decreasing *Bcl-2, PTGS2* and *iNOS* gene expression (anti-apoptotic gene). Our study further reinforces the potential benefit of combination of both drugs in cancer treatment and provides novel mechanistic insight into their antiproliferative role.

### Supplementary Information

**Supplementary Material 1.**

## Data Availability

All data generated or analyzed during this study are included in this published article. If detailed data are required, they can contact the correspondence of the study, EI. Salim (Email address: elsayed.salim@science.tanta.edu.eg).

## Abbreviations

AMPK: Adenosine monophosphate-activated protein kinase
CI: Combination index
DAVID: Database of Annotation, Visualization, and Integrated Discovery
DPPH: 2,2-Diphenyl-1-picrylhydrazyl
FACS: Fluorescence-activated cell sorting
GO: Gene Ontology
GSH: Reduced glutathione
HCC: Hepatocellular carcinoma
IC_50_: Half-maximal inhibitory concentration
KEGG: Kyoto Encyclopedia of Genes and Genomes
mTOR: Mammalian target of rapamycin
PPAR: Peroxisome proliferator-activated receptor
ROS: Reactive oxygen species
RNS: Reactive nitrogen species
RPMI: Roswell Park Memorial Institute
