# Supplementary material for: Molecular interactions between metformin and D-limonene inhibit proliferation and promote apoptosis in breast and liver cancer cells
Source: BMC Complement Med Ther. 2024 May 6;24:185. doi: 10.1186/s12906-024-04453-x (PMC11071183; doi:10.1186/s12906-024-04453-x)
Supplement: Supplementary file 1 — Supplementary Material 1. [file 12906_2024_4453_MOESM1_ESM.docx]

**Supplementary Table 1:** Primer Sequences of *Bax, Bcl-2, P53, PTGS2 (COX2), iNOS* genes, and internal housekeeping control gene.

| **Size of PCR product** | **Primer’s sequences** | **Genes** |
| --- | --- | --- |
| 187 bp | F: 5ˋ - GTCTTTTTCCGAGTGGCAGC-3ˋ | ***PTGS2(COX2)*** |
|  | R: 5ˋ-CCACTGCAGGGGGTTGATAC-3ˋ |  |
| 136 bp | F: 5ˋ-CCAGGACGCATCCACCAAGAAGC-3ˋ | ***Bax*** |
|  | R: 5ˋ-TGCCACACGGAAGAAGACCTCTCG-3ˋ |  |
| 118 bp | F: 5’-GGATGACTTCTCTCGTCGCTACCGT-3’ | ***Bcl-2*** |
|  | R: 5-’ATCCCTGAAGAGTTCCTCCACCAC-3’ |  |
| 106 bp | F: 5ˋ-CTACTAAGGTCGTGAGACGCTGCC-3ˋ | ***P53*** |
|  | R: 5ˋ-TCAGCATACAGGTTTCCTTCCACC-3 |  |
| 172bp | F:5′-GGAATCGATATGAACGCCAGC-3′ | ***iNOS*** |
|  | R:5′-CCTTGTTAAGTGGCGATGGG-3′ |  |
| 209 bp | F: 5ˋ-ATGGAGAAGGCTGGGGCTCACCT -3ˋ | ***GAPDH*** |
|  | R: 5ˋ-AGCCCTTCCACGATGCCAAAGTTGT -3ˋ |  |

F: Forward; R: Reveres; bp: Base pairs.

**Supplementary Table 2:** Enriched KEGG pathways of metformin and *D*-limonene-related targets.

| **Count** | **Genes** | **Pathway** |
| --- | --- | --- |
| 8 | *HSD11B2, HSD11B1, HSD17B3, CYP19A1, CYP17A1, SRD5A1, SRDA2, UGT2B7* | **Steroid hormone biosynthesis** |
| 6 | *ALOX5, PTGS2, PTGS1, CYP2C19, PLA2G1B, PTGES* | **Arachidonic acid metabolism** |
| 11 | *SCD, NR1H3, RXRG, RXRA, FABP1, FABP4, PPARA, FABP3, FABP5, PPARG, PPARD* | **PPAR signaling pathway** |
| 15 | \| *PTGER1, CHRM2, NOS3, NOS2, NOS1, ADORA2A, HTR5A, HTR6, HTR7, HTR2A, HTR2C, HTR2B, ADRA1D, ADRA1B, ADRA1A* \| \| --- \| | **Calcium signaling pathway** |
| 28 | \| *PTGER2, HRH4, ADRA2C, ADRA2A, ADRA2B, TRPV1, ADORA1, ADORA3, F2, HTR1D, HTR1B, HTR1A, CNR2, CNR1, HRH3, NR3C1* \| \| --- \| | **Neuroactive ligand-receptor interaction** |
| 7 | \| *PRKCH, MAPK3* \| \| --- \| | **Vascular smooth muscle contraction** |
| 5 | \| *PTPN6, PTPRF, ACP1, PTPN1* \| \| --- \| | **Adherens junction** |
| 5 | \| *ITGB3, ITGAV* \| \| --- \| | **Regulation of actin cytoskeleton** |
| 13 | \| *RARB, PARA, AR, GLI1, GLI2* \| \| --- \| | **Pathways in cancer** |

**Supplementary Table 3: GO information data.**

| **no** | **Function Pathway** | **Degree** |
| --- | --- | --- |
| 1 | NEUROACTIVE_LIGAND_RECEPTOR_INTERACTION | 28 |
| 2 | CALCIUM_SIGNALING_PATHWAY | 15 |
| 3 | PATHWAYS_IN_CANCER | 13 |
| 4 | PPAR_SIGNALING_PATHWAY | 11 |
| 5 | STEROID_HORMONE_BIOSYNTHESIS | 8 |
| 6 | VASCULAR_SMOOTH_MUSCLE_CONTRACTION | 7 |
| 7 | ARACHIDONIC_ACID_METABOLISM | 6 |
| 8 | SMALL_CELL_LUNG_CANCER | 6 |
| 9 | ADHERENS_JUNCTION | 5 |
| 10 | REGULATION_OF_ACTIN_CYTOSKELETON | 5 |

**Supplementary Table 4:** Combination index (CI) data. **A)** Metformin + *D*-limonene combination (2:1) on HepG-2 cells for 48 hrs. **B)** Metformin + *D*-limonene combination (2:1) on MCF-7 cells for 48 hrs.

| **A**  **Fa** | **Single-dose** | | **Metformin + *D-*limonene combination**  **(HepG-2 cells)** | | | | |
| --- | --- | --- | --- | --- | --- | --- | --- |
|  | **Met** | **Lim** | **CI** | **Combined dose** | **Met + Lim** | **DRI** | |
|  |  |  |  |  |  | **Met** | **Lim** |
| 0.25 | 19.12 | 7.12 | 0.146 | 1.79 | 1.2+0.60 | 16.02 | 11.94 |
| 0.5 | 45.4 | 16.83 | 0.143 | 4.2 | 2.8+1.4 | 16.4 | 12.14 |
| 0.75 | 107.8 | 39.8 | 0.140 | 9.66 | 6.4+3.2 | 16.7 | 12.4 |
| 0.9 | 255.8 | 93.97 | 0.138 | 22.43 | 14.95+7.5 | 17.1 | 12.6 |
| 0.95 | 460.5 | 168.6 | 0.136 | 39.79 | 26.5+13.3 | 17.4 | 12.7 |
| **B**  **Fa** | **Single-dose** | | **Metformin + *D-*limonene combination**  **(MCF-7 cells)** | | | | |
|  | **Met** | **Lim** | **CI** | **Combined dose** | **Met + Lim** | **DRI** | |
|  |  |  |  |  |  | **Met** | **Lim** |
| 0.25 | 33.69 | 19.6 | 0.71 | 19.3 | 12.9+6.43 | 2.62 | 3.1 |
| 0.5 | 103.7 | 51.8 | 0.455 | 35.4 | 23.6+11.8 | 4.4 | 4.4 |
| 0.75 | 319.4 | 136.6 | 0.29 | 64.9 | 43.24+21.6 | 7.4 | 6.3 |
| 0.9 | 983.3 | 360.4 | 0.19 | 118.9 | 79.23+39.61 | 12.4 | 9.1 |
| 0.95 | 2112.75 | 696.9 | 0.14 | 179.46 | 119.6+59.8 | 17.7 | 11.7 |

**Fa:** default effect level; **CI**: combination index; **DRI**: dose-reduction index.

**Supplementary Table 5:** qRT-PCR data for HepG-2 cells **(A)** and MCF-7 cells **(B)**.

| **A**  **Groups** | ***BAX RC***  (2^- ΔΔCT^) | ***Bcl-2 RC***  (2^- ΔΔCT^) | ***P53 RC***  (2^- ΔΔCT^) | ***PTGS2 RC***  (2^- ΔΔCT^) | ***iNOS RC***  (2^- ΔΔCT^) |
| --- | --- | --- | --- | --- | --- |
| **Untreated normal cells** | 1 ± 0.00 | 1 ± 0.00 | 1 ± 0.00 | 1 ± 0.00 | 1 ± 0.00 |
| **Untreated HepG2 cells** | 0.62 ± 0.015 | 3.6 ± 0.05 | 0.69 ± 0.03 | 4.5 ± 0.05 | 7.7 ± 0.019 |
| **Metformin** | 3.7 ± 0.015* | 0.72 ± 0.002* | 5.23 ± 0.019 | 0.54 ± 0.021 | 0.42 ± 0.011 |
| ***D*-limonene** | 5.4 ± 0.025 | 0.22 ± 0.003* | 8.2 ± 0.0126 | 0.72 ± 0.007* | 0.64 ± 0.011 |
| **Met : lim combination (1:2)** | 6.1 ± 0.067 | 0.53 ± 0.004* | 6.89 ± 0.09 | 0.24±0.015 | 0.19±0.014 |
| **B**  **Groups** | ***BAX RC*** (2^- ΔΔCT^) | ***Bcl2 RC***  (2^- ΔΔCT^) | ***P53 RC***  (2^- ΔΔCT^) | ***PTGS2 RC***  (2^- ΔΔCT^) | ***iNOS RC***  (2^- ΔΔCT^) |
| **Untreated normal cells** | 1 ± 0.00 | 1 ± 0.00 | 1 ± 0.00 | 1 ± 0.00 | 1 ± 0.00 |
| **Untreated MCF-7 cells** | 0.5 ± 0.01* | 2.8 ± 0.15* | 0.46 ± 0.02* | 3.3 ± 0.12* | 10.0 ± 0.10* |
| **Metformin** | 5.4 ± 0.09* | 0.44 ± 0.005* | 6.79 ± 0.13* | 0.58 ± 0.014* | 0.53 ± 0.009** |
| ***D*-limonene** | 3.9 ± 0.1* | 0.69 ± 0.01* | 5.28 ± 0.05* | 0.66 ± 0.019* | 0.69 ± 0.014* |
| **Met : lim combination (1:2)** | 7.1 ± 0.06* | 0.55 ± 0.015* | 8.7 ± 0.11* | 0.31 ± 0.01* | 0.31 ± 0.009** |

**RC**: Relative gene expression normalized to the endogenous housekeeping gene.
